# Early assessment of treatment response in primary biliary cholangitis: key to timely management

**DOI:** 10.1186/s12876-025-04138-w

**Published:** 2025-08-09

**Authors:** Tomas Koky, Sylvia Drazilova, Martin Janicko, Dominika Toporcerova, Jakub Gazda, Peter Jarcuska

**Affiliations:** 1 Department of Internal Medicine, Faculty of Medicine, PJ Safarik University, L Pasteur University Hospital, Kosice, Slovakia; 2 Department of Internal Medicine, Hospital Poprad, Poprad, Slovakia

**Keywords:** Primary biliary cholangitis, Ursodeoxycholic acid, Treatment response, Second line treatment, Liver decompensation, Prognosis

## Abstract

**Aim:**

The aim of our study was to explore predictive factors associated with compete biochemical response (CBR) in primary biliary cholangitis (PBC) patients treated with ursodeoxycholic acid (UDCA) at month 12 and at last check-up; CBR was defined as both normal bilirubin and ALP levels. We also evaluated hepatic decompensation and prognosis during UDCA treatment.

**Methods:**

We conducted a multicenter retrospective study of PBC patients. We enrolled patients with PBC before the beginning of UDCA treatment (13–15 mg/kg body weight per day) between 1999 and 2024 in 2 hepatology centers in Eastern Slovakia.

**Results:**

We enrolled 155 patients in the final analysis, 147 women and 8 men, mean age at diagnosis 57 ± 15 years, the median follow-up was 10 ± 8 years. 29 patients (18.7%) had cirrhosis at diagnosis. Hepatic decompensation occurred in 12 patients during follow-up (7.7%; 95%CI 4–13%). 114 patients (73.5%; 95% CI 66–80%) achieved response to treatment according to Toronto criteria at month 6; CBR after 12 months of treatment was achieved by 41.3% (95% CI 33–50%) of patients; 51 patients (32.9%; 95% CI 26–41%) achieved CBR at last check-up. The OR for achieving CBR at month 12 in Toronto responders at month 6 was 46.36 (95%CI 6.147-349.646); *p* < 0.001. Of the patients who achieved a response defined by Toronto criteria at month 6, 44.7% of patients achieved CBR at last check-up and 55.3% did not. Out of the patients who did not achieve Toronto at month 6, none achieved CBR at last check-up (0%). Treatment response by Toronto criteria at month 6, CBR at month 12 and absence of liver cirrhosis predicted CBR at last check-up. The odds of decompensation were about 90% lower in patients who achieved treatment response. No case of hepatic decompensation or liver-related mortality during follow-up was observed in patients with CBR at last check-up.

**Conclusion:**

The assessment of treatment response at month 6 is very accurate predictor of complete biochemical response throughout the course of the disease. For non-responders to UDCA treatment, the addition of second line PBC treatment is indicated. For patients who have not met Toronto criteria at month 6 of UDCA treatment, the addition of second line PBC therapy should be considered.

## Introduction

Primary biliary cholangitis (PBC) is a non-suppurative autoimmune liver disease that leads to destruction of the bile ducts and liver fibrosis. In a small proportion of patients the disease progresses to liver cirrhosis [1, 2]. PBC is a rare disease, it occurs more frequently in women and the prevalence of the disease has increased in European countries in recent years [3]. To diagnose a case of PBC, 2 of 3 criteria need to be met:

- increased ALP above the upper limit of the norm lasting for at least 6 months,
- AMA M2 positivity in a titer of at least 1:40; in case of AMA negativity, specific ANA positivity (anti-sp100 or anti-gp210),
- histological findings consistent with PBC [1].

Although AMA positivity is typical for the diagnosis of PBC, ANAs are positive in 50% of AMA-positive PBC patients and in 85% of AMA-negative PBC patients [4]. Anti-gp210, anti-sp100 and anti-sp140 are the most common ANA subtypes in PBC [5–7].

An overlap of PBC with AIH or PSC can be observed in clinical practice, but to confirm the overlap, patient must fulfill the diagnostic criteria of both diseases. Patients with overlap syndrome have a worse prognosis than patients with PBC alone [8, 9]. PBC has multiple extrahepatic clinical manifestations that reduce quality of life. The most common of these are pruritus, fatigue, osteoporosis and dyslipoproteinemia [10].

The goal of PBC treatment is to achieve a biochemical, histological and clinical remission. Effective treatment of PBC should improve patients’ prognosis and quality of life. The drug of choice for PBC patients is UDCA at a dose of 13–15 mg/kg bw per day [1]. There are many scoring systems to evaluate the treatment response [11]. Patients who achieve a biochemical response to treatment have lower risk of hepatic decompensation compared to nonresponders to UDCA therapy [12]. UDCA therapy also improves transplant-free survival in PBC patients [13]. Second line PBC treatment for UDCA non-responders is available, it can improve a clinical course of the disease [14]. Second line treatment is generally indicated in nonresponders after one year of treatment. However, in some patients the disease may progress rapidly and earlier add-on second line therapy should be considered.

The overall aim of the presented research is to identify and refine early predictors of the treatment response to allow the prompt inclusion of potential non-responders into second line treatment or clinical trials.

The objectives of this study were to evaluate the accuracy of Toronto treatment response criteria at month 6 of UDCA treatment for the prediction of CBR at the end of follow-up and after 12 months of treatment and to evaluate the predictive accuracy of CBR12 for the prediction of complete biochemical response after long term follow-up. Further aims were to describe the risk of mortality and decompensation in groups stratified by treatment response.

## Patients and methods

We conducted a multicenter retrospective study of PBC patients. We enrolled patients with PBC treated with UDCA (13–15 mg/kg body weight per day) between 1999 and 2024 in 2 hepatology centers in Eastern Slovakia (2nd Department of Internal Medicine, PJ Safarik University, Faculty of Medicine and L Pasteur University Hospital, Kosice; and Department of Internal Medicine, Hospital Poprad). The diagnosis of PBC was confirmed at the first visit according to EASL criteria [1]. The exclusion criteria for enrolment in the study were:

- Overlap syndrome PBC with AIH or PSC at diagnosis.
- Overlap with chronic hepatitis B or C
- UDCA, OCA or fibrate use prior to study enrolment.
- Use of medications that could affect UDCA treatment response UDCA treatment response (corticosteroids, immunosuppressants, hormonal treatment, chemotherapy etc.)
- Lack of compliance with UDCA treatment during follow-up.
- Concomitant advanced liver diseases.
- Liver transplantation prior to inclusion.
- Duration of UDCA treatment less than 18 months during follow-up.
- Incomplete data after 6 months of UDCA treatment and at last follow-up.
- Advanced systemic disease, malignancy (except localized skin cancer).

At inclusion following variables were collected from the patient documentation: total bilirubin, AST, ALT, GGT, ALP, albumin, platelets, prothrombin time (INR) and CRP. Patients were followed every 6 months as is the standard of care for PBC both centers. At follow-up visits, further values of total bilirubin and ALP were collected.

Since hepatologists evaluate biochemical response at the actual check-up, we decided to evaluate treatment response not only at 6 and 12 months, but also at the last check-up, which appears to be the simplest criterion to assess long-term response to UDCA treatment applicable in real clinical practice. After 6 months of UDCA treatment, we evaluated therapeutic response according to the modified Toronto criteria; defined as ALP ≤ 1.67 × ULN and total bilirubin ≤ 2 × ULN [15]. After 12 months of UDCA treatment and at last check-up, we assessed CBR (ALP ≤ ULN and total bilirubin ≤ ULN).

Primary outcome was defined as the proportion of patients that achieved CBR, defined as normal bilirubin and normal ALP levels, at last check-up.

Secondary outcomes were:

a the proportion of patients that achieved CBR after 12 months of treatment.
b Proportion of patients with hepatic decompensation during follow-up.

### Statistical analysis

The values of total bilirubine, AST, ALT, GGT and ALP are presented as multiples of the upper limit of norm (denoted as %ULN). Values are presented as mean. standard deviation. standard error of mean. median and interquartile range for interval variables and absolute and relative counts for categorical. Normality of distribution is tested by Shapiro Wilk test. Differences in distributions are tested by T-test for normally distributed variables and MannWhitney for others. Differences in categorical variables are tested by Chi-squared test. Fisher exact test was used in calculation of mortality and decompensation proportions, where low expected counts were observed. Unadjusted odds and ORs are calculated from 2 × 2 tables. Models with multiple predictors. constructed by multivariate logistic regression. are used to adjust the main predictors of interest to other relevant confounders. Survival was evaluated by Kaplan-Mayer curves and differences evaluated by log rank test.

## Results

Between 1999 and 2024, we followed 216 patients with PBC. 61 patients were not included in the final analysis:

- 8 patients due to overlap PBC with AIH or PSC.
- 1 patient used corticosteroids at diagnosis from non-hepatic indication.
- 1 patient used immunosuppressive drugs at diagnosis from non-hepatic indication.
- 3 patients started UDCA treatment at another department before referral to one of our liver centers.
- 1 patient was treated with fibrate due to hypertriglyceridemia at diagnosis.
- 1 patient was treated by hormonal therapy at diagnosis, which exacerbated cholestasis
- 3 patients were non-compliant to UDCA during follow-up
- 25 patients had incomplete data after 6 months of UDCA treatment.
- 18 patients were followed up for less than 18 months.

Forty-three patients received second line treatment during follow-up: 37 fenofibrate and 6 OCA. CBR at last check-up was assessed before the initiation of second line treatment in these patients. 3 patients underwent liver transplantation during follow-up. In these patients, CBR at last check-up was assessed at the last visit before liver transplantation.

A total of 155 patients were evaluated. 8 males and 147 females. Mean age at diagnosis was 57 years. 29 patients (18.7%) had cirrhosis. The median follow-up period was 10 years, interquartile range of follow-up 8 years. During follow-up 19 patients died or underwent transplantation (12%; 95%CI 7.5- 18.5%), of whom liver-related mortality or transplantation was 3.2% (5 patients); 95% CI 1.1–7.4%. Liver cirrhosis decompensation occurred in 12 patients (7.7%; 95%CI 4–13%).

### Primary outcome – complete biochemical response at last check-up

Biochemical response to UDCA treatment at month 6 was evaluated in all patients. 114 patients (73.5%; 95% CI 66–80%) achieved response to treatment according to Toronto at month 6. The basic characteristics of the cohort are shown in Table 1.

**Table 1:** Description of the study cohort

	Mean	Standard Error of Mean	Standard Deviation	Median	IQR
Age at diagnosis (years)	57	1	11	56	16.00
Follow-up (years)	10	0	5	10	8.00
Total bilirubine at Dx (%ULN)	0.87	0.09	1.17	0.60	0.37
ALP at Dx (%ULN)	2.35	0.15	1.89	1.69	1.32
GGT at Dx (%ULN)	4.56	0.45	5.20	2.66	4.23
ALT at Dx (%ULN)	1.70	0.12	1.54	1.28	1.34
AST at Dx (%ULN)	1.50	0.09	1.06	1.12	1.12
Albumin at Dx (g/L)	41.95	0.44	5.24	42.30	5.25
Platelets at Dx (x10^9^/L)	236.1	6.4	76.5	232.0	103.00
INR at Dx	0.99	0.01	0.12	0.97	0.10
AST/ALT at Dx	1.08	0.05	0.62	0.93	0.47
ALT/ALP at Dx	0.56	0.05	0.60	0.38	0.53
CRP at Dx (mg/L)	11.01	2.89	26.96	4.35	6.90
%ULN – multiple of the upper limit of norm, IQR – interquartile range					

Primary outcome was the achievement of CBR at last check-up. 51 patients (32.9%; 95% CI 26–41%) achieved CBR at last check-up. We compared the accuracy of the two main predictors of this response namely Toronto at month 6 and CBR at month 12.

Of the patients who achieved a response defined by Toronto criteria at month 6, 44.7% of patients achieved CBR at last check-up and 55.3% did not. Out of the patients who did not achieve Toronto at month 6, none achieved CBR at last check-up (0%), therefore the OR could not be calculated. The odds that patients who reached Toronto at month 6 will have a CBR at the last checkup is 81%; *P* < 0.001.

Of the patients who had CBR at month 12, 72.6% achieved CBR at last check-up. Only 4 patients who did not have CBR month 12 achieved CBR at last check-up (4.5%). The OR is 55.59; 95%CI 17.6-175.2; *p* < 0.001.

Of the patients who had baseline cirrhosis, only 10.3% (3 patients) achieved CBR at last check-up. 48 (38.1%) patients without baseline liver cirrhosis achieved CBR at last check-up. The OR was 0.19; 95%CI 0.05–0.65; *p* = 0.002.

The differences in selected parameters according to the achievement of CBR at the last check-up are summarized in Table 2. Patients who achieved CBR at last check-up were borderline significantly older. They had lower basal, 6 and 12-month ALP levels, had borderline lower ALT and AST. There was no difference in bilirubin levels at any of the follow-up visits.

**Table 2:** Differences in selected parameters according to the achievement of complete biochemical response at the last check-up

	Complete biochemical response at last check up				
	Not achieved (*n* = 104)		Achieved (*n* = 51)		
	Mean	Standard Deviation	Mean	Standard Deviation	P
Age at diagnosis (years)	56	10	60	13	**0.035**
Total bilirubin at Dx (%ULN)	1.00	1.39	0.60	0.19	0.06
ALP at Dx (%ULN)	2.70	2.17	1.65	0.76	**< 0.001**
GGT at Dx (%ULN)	4.89	5.94	3.91	3.27	0.745
ALT at Dx (%ULN)	1.89	1.75	1.31	0.86	**0.049**
AST at Dx (%ULN)	1.65	1.18	1.19	0.64	**0.011**
Albumin at Dx (g/L)	41.80	5.83	42.26	3.80	0.881
Platelets at Dx (x10^9^/L)	241.1	75.2	225.0	78.9	0.248
INR at Dx	0.99	0.14	0.99	0.07	0.345
AST/ALT at Dx	1.06	0.65	1.11	0.54	0.648
ALT/ALP at Dx	0.56	0.66	0.58	0.45	0.306
CRP at Dx (mg/L)	14.59	33.41	4.85	3.27	0.163
ALP at 6 months (%ULN)	1.85	1.41	0.95	0.29	**< 0.001**
Total bilirubin at month 6 (%ULN)	0.81	1.02	0.55	0.22	0.392
ALP at 12 months (%ULN)	1.68	1.19	0.80	0.18	**< 0.001**
Total bilirubin at month12 (%ULN)	0.84	1.09	0.56	0.24	0.793

%ULN – multiples of the upper limit of norm, Dx - diagnosis

To adjust the predictors to all the other variables that were significantly different between the groups we created multivariate logistic regression models (Table 3). Because of the obvious correlation between two main predictors (Toronto at month 6 and CBR at month 12) two models were fitted and their model fits compared. Other significant variables (age, cirrhosis, AST and ALT levels) remained the same in each of the models.

**Table 3:** Multivariate logistic regression models with the main predictors adjusted for all other variables significantly different between patients who did and did not achieve primary outcome

	Sig.	Exp(B)	95% C.I.for EXP(B)	
			Lower	Upper
Model 1 - main predictor - response defined by Toronto at month 6				
Cirrhosis	0.021	0.203	0.052	0.789
Age at Dx (years)	0.051	1.039	1.000	1.079
ALT at Dx (%ULN)	0.738	1.124	0.567	2.226
AST at Dx (%ULN)	0.708	0.834	0.322	2.157
Toronto resonse at month 6	N/A	N/A	N/A	N/A
Constant	0.997	0.000		
Model 2 - main predictor – Complete biochemical response at month 12				
Cirrhosis	0.239	0.347	0.059	2.022
Age at Dx (years)	0.126	1.037	0.990	1.086
ALT at Dx (%ULN)	0.177	0.601	0.287	1.259
AST at Dx (%ULN)	0.430	1.543	0.526	4.532
CBR at month 12	< 0.001	61.156	17.791	210.218
Constant	0.002	0.008		

Model 1 includes treatment response defined by Toronto at month 6 as a predictor of CBR at last check-up with sensitivity 100% (95%CI 93–100%); specificity 39% (95%CI 30–49%) and accuracy 59% (95%CI 51–67%). The predictor itself is uninterpretable due to quasi-complete separation (all patients who did not achieve Toronto at month 6, did not achieve CBR at last check-up). The overall model fit (R squared) is 0.386. Out of the other predictors, only the presence of cirrhosis is significant.

Model 2 includes CBR at month 12 as a main predictor of CBR at last check-up with sensitivity 92% (95%CI 80–98%); specificity 83% (95%CI 74–90%) and accuracy 86% (95% CI 79–91%). This predictor is the only independent predictor of outcome in the model, and the overall model fit (R2 is 0.641) is excellent - explaining over 60% of the variability in outcome. Comparison of the sensitivity, specificity and accuracy of the two models is compared in Table 4. CBR at month 12 is a significantly more accurate predictor of CBR at last check-up than Toronto at 6 months. Statistical significance is highlighted by the fact that confidence intervals do not overlap.

### Secondary outcome – Complete biochemical response at month 12 of the treatment

CBR after 12 months of treatment was achieved by 41.3% (95% CI 33–50%) of patients. The main predictor of interest was response to the treatment according to Toronto at month 6. 55% of patients who achieved a Toronto response at month 6 also achieved a CBR at month 12. However, almost all patients (97.4%) who did not achieve Toronto response at month 6 did not achieve a CBR at month 12. The OR for achieving CBR at month 12 in Toronto responders at month 6 is 46.36 (95%CI 6.147-349.646); *p* < 0.001.

Patients with cirrhosis had a significantly lower chance of achieving CBR at month12, OR 0.263, 95%CI 0.094–0.740; *p* = 0.008. Only 5 patients (18.5%) with cirrhosis achieved CBR at month 12. 22 (81.5%) patients with cirrhosis did not achieve CBR at month 12. Table 4 shows the differences in other parameters between patients who did and did not achieve secondary outcome. Patients who achieved CBR at month 12 had borderline lower baseline total bilirubine, significantly lower ALP and AST and significantly lower total bilirubin and ALP at month 6 of treatment.

**Table 4:** Differences in selected parameters according to the achievement of complete biochemical response at month 12 of the treatment

	**Complete biochemical response at month 12 of the treatment**				
	Not achieved (*n*=88)		Achieved (*n*=62)		
	Mean	Standard Deviation	Mean	Standard Deviation	P
**Age at diagnosis (years)**	56	10	59	12	0.132
**Total bilirubin at Dx (%ULN)**	1.02	1.48	0.66	0.40	**0.05**
**ALP at Dx (%ULN)**	2.77	2.21	1.69	0.96	**<0.001**
**GGT at Dx (%ULN)**	4.70	5.04	4.23	5.55	0.317
**ALT at Dx (%ULN)**	1.72	1.18	1.53	1.40	0.106
**AST at Dx (%ULN)**	1.59	0.95	1.25	0.84	**0.004**
**Albumin at Dx (g/L)**	42.17	4.27	41.86	6.36	0.988
Platelets at Dx (x10^9^/L)	237.8	73.5	235.5	80.4	0.860
**INR at Dx **	1.01	0.14	0.98	0.08	0.746
**AST/ALT at Dx **	1.07	0.62	1.08	0.62	0.659
**ALT/ALP at Dx **	0.46	0.36	0.68	0.78	0.108
**CRP at Dx (mg/L)**	15.82	36.76	5.97	4.73	0.552
**ALP at 6 months (%ULN)**	17.23	21.65	11.51	6.47	**0.024**
**Total bilirubin at month 6 (%ULN)**	1.93	1.44	0.96	0.33	**<0.001**

%ULN – multiples of the upper limit of norm, Dx - diagnosis

A multivariate regression model was constructed to evaluate the effect of individual variables that were significantly different between groups based on the achievement of the CBR at month 12. Bilirubin and ALP were not entered into the model because of significant collinearity with the main predictor (Toronto response at month 6) - Table 5. The model shows that the Toronto response at month 6 is the only significant predictor of CBR at month 12, although the presence of cirrhosis is marginally close to statistical significance. The overall model fit (R2) is 0.366.

**Table 5:** Multivariate logistic regression model used to adjust predictor (Response at month 6 according to Toronto criteria) to other relevant variables

	Sig.	Exp(B)	95% C.I.for EXP(B)	
			Lower	Upper
Cirrhosis	0.062	3.012	0.946	9.592
AST at Dx (%ULN)	0.475	1.227	0.699	2.155
Response Toronto at month 6	< 0.001	43.187	5.52	337.9
Total bilirubin at Dx (%ULN)	0.100	0.433	0.160	1.173
Constant	0.006	0.045		

### Secondary outcome – mortality and decompensation

**Fig. 1:**
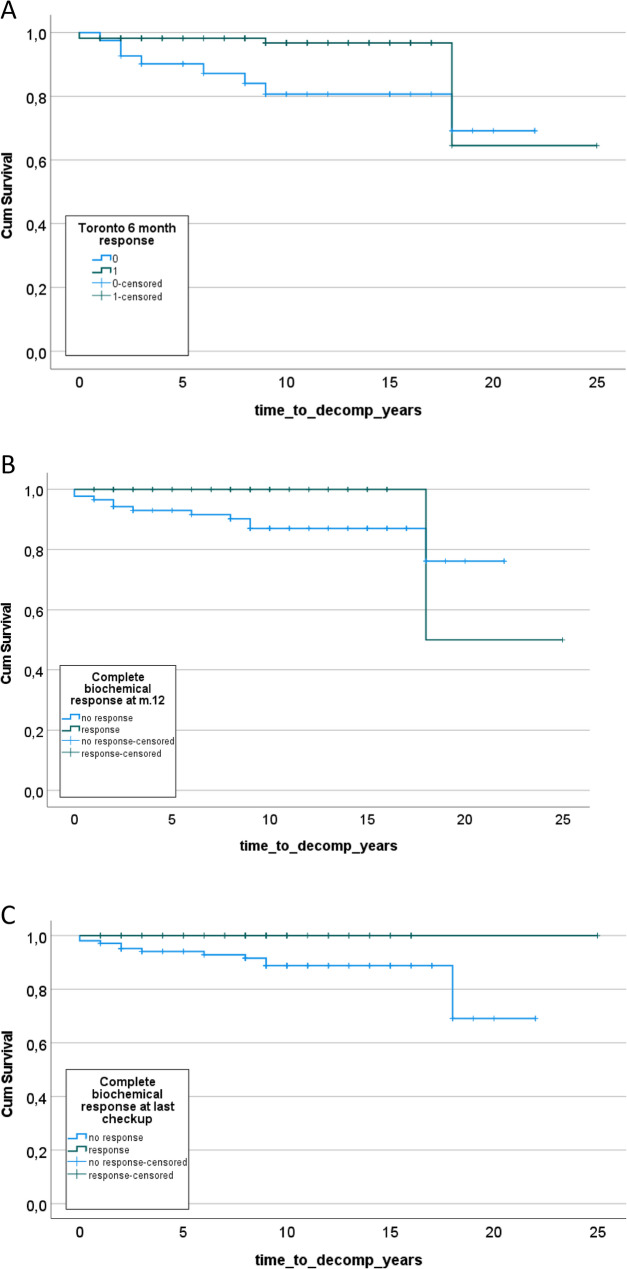
Probability of liver cirrhosis decompensation during follow-up in groups with treatment response defined by Toronto criteria at month 6 (panel **A**), *p*=0.011; treatment response defined by complete biochemical response at month 12 (panel **B**), *p*=0.038; treatment response defined by complete biochemical response at last check-up (panel **C**), *p*=0.026

Decompensation of liver cirrhosis was observed in 12 patients (7.7%. 95% CI 4–13%). the proportion of patients with decompensation stratified by response according Toronto criteria at month 6, CBR at month 12 and CBR at the end of follow-up is shown in Table 6. As expected, patients who achieved treatment response defined by any criteria had significantly lower rate of decompensation. The probability of liver cirrhosis decompensation is shown in Fig. 1. The odds of decompensation were about 90% lower in patients who achieved treatment response. No patient who had CBR at the last check-up developed decompensation, thus OR could not be calculated.

**Table 6:** Proportions of patients who developed cirrhosis decompensation stratified by the achievement of treatment response defined by Toronto at month 6, complete biochemical response at month 12 and at last check-up

	No Decompensation		Decompensation					
	Count	%	Count	N %		OR	95%CI	P
Toronto response at month 6	no response	33	80.5%	8	19.5%	0.15	0.04–0.53	0.003
	response	110	96.5%	4	3.5%			
Complete response at month 12	no response	77	87.5%	11	12.5%	0.11	0.01–0.91	0.016
	response	61	98.4%	1	1.6%			
Complete response at last check-up	no response	92	88.5%	12	11.5%	N/A	N/A	0.009
	response	51	100.0%	0	0.0%			

Nineteen patients died or underwent liver transplantation during follow-up (12%; 95%CI 7.5- 18.5%). The proportion of patients who died or underwent liver transplant stratified by Toronto 6 response, CBR at month 12 and CBR at last check-up is shown in Table 7. The only significant predictor of survival was the response to treatment according to Toronto criteria at month 6.

**Table 7:** Proportions of patients who died or underwent liver transplantation stratified by the achievement of treatment response defined by Toronto at month 6, complete biochemical response at month 12 and at last check-up

		**Surviving**		**Died or transplanted**				
		Count	%	Count	%	OR	95%CI	p
**Toronto response at month 6 **	no response	32	78%	9	22%	0.34	0.13-0.91	0.027
	response	104	91.2%	10	8.8%			
**Complete response at month 12**	no response	73	83.0%	15	17.0%	0.34	0.11-1.07	0.055
	response	58	93.5%	4	6.5%			
**Complete response at last check-up**	no response	88	84.6%	16	15.4%	0.34	0.1-1.24	0.09
	response	48	94.1%	3	5.9%			

The proportion of patients who died due to liver specific causes or underwent liver transplantation was 3.2% (5 patients), 95% CI 1.1–7.4%. The proportion of patients who died or underwent liver transplant stratified by Toronto response at month 6, CBR at month 12 and CBR at last check-up is shown in Table 8.

**Table 8:** Proportions of patients who died due to liver specific causes or underwent transplantation stratified by the achievement of treatment response defined by Toronto at month 6, complete biochemical response at month 12 and at last check-up

		**Surviving**		**Died or transplanted**				
		Count	N %	Count	N %	OR	95%CI	p
**Toronto response at month 6 **	no response	37	90.2%	4	9.8%	0.08	0.009-0.76	0.018
	response	113	99.1%	1	0.9%			
**Complete response at month 12**	no response	83	94.3%	5	5.7%	N/A	N/A	0.077
	response	62	100.0%	0	0.0%			
**Complete response at last check-up**	no response	99	95.2%	5	4.8%	N/A	N/A	0.172
	response	51	100.0%	0	0.0%			

## Discussion

The optimal goal of UDCA treatment for PBC patients is to achieve CBR, which is defined as follows: bilirubin and ALP levels ≤ ULN. Patients with long-term CBR have a better prognosis compared to patients who do not achieve CBR [16]. The primary outcome of our study was the proportion of patients that achieved CBR at last check-up. We investigated the association between biochemical response according Toronto criteria at month 6 and achievement of CBR at last check-up. Approximately 1/3 of all UDCA-treated patients and 45% of responders to UDCA therapy according to Toronto criteria at month 6 achieved CBR at last check-up. However, no PBC patients who did not have a biochemical response by Toronto criteria at month 6 achieved CBR at last check-up. In multivariate analysis, absence of liver cirrhosis before treatment initiation, biochemical response according to Toronto criteria at month 6, and CBR at month 12 predicted achievement of CBR at last check-up. No case of hepatic decompensation or liver-related mortality during follow-up was observed in patients with CBR at last check-up.

Biochemical response to UDCA treatment according Toronto criteria at month 6 predicted CBR after 1 year of UDCA treatment (OR 43.187; 95 CI 5.52–337.9).

Patients who achieved a biochemical response according to the Toronto criteria at month 6 had a significantly lower chance of liver decompensation and significantly better liver-specific survival than non-responders.

For non-responders to UDCA treatment, the addition of second line PBC treatment is indicated. Patients treated with OCA were significantly more likely to have a biochemical response in the POISE registration study compared to placebo [17]. Approximately half of all OCA-treated patients had a long-term biochemical response, and histological findings regressed in some patients [18, 19]. A serious side effect of OCA treatment is pruritus [17]. Another limitation of OCA treatment is its high cost. OCA therapy is associated with a high risk of hepatic decompensation or death in PBC patients with advanced liver cirrhosis and it is contraindicated in these patients [2, 20]. Long-term OCA treatment was discredited by the confirmatory COBALT study, in which patients treated with OCA did not have a better prognosis compared to placebo [21].

Bezafibrate treatment improves biochemical parameters in PBC patients, the treatment is cheap and relatively safe [22]. The limitation of bezafibrate treatment is that it is not registered for PBC patients and is not available in many countries. When bezafibrate is unavailable, fenofibrate or ciprofibrate can be used in second line PBC treatment [23, 24]. Budesonide improves biochemical but not histological findings in PBC patients [25].

Several drugs are being studied in second-line PBC treatment in clinical trials:

- PPAR agonists.
- non-steroidal FXR agonists.
- FGF-19 modulators.
- inhibition of NOX1 and NOX4.
- nor-UDCA [14].

PPAR agonists are the most promising drugs for second line therapy in the future. Seladelpar is a PPARδ agonist that demonstrated significant biochemical improvement and reduction of pruritus compared to placebo in a Phase III clinical trial [26]. PPARα/δ agonist elafibranor led to biochemical improvement compared to placebo in Phase III clinical trial, drug is relatively safe [27]. Another prospective PPAR agonist is saroglitazar [28]. Non-steroidal FXR agonists cilofexor and tropifexor improved cholestasis, but the limiting factor in their use is pruritus [14]. FGF-19 modulator aldafermin may also be a promising drug for second-line PBC treatment, which improves cholestasis without pruritus worsening [29].

**Fig. 2:**
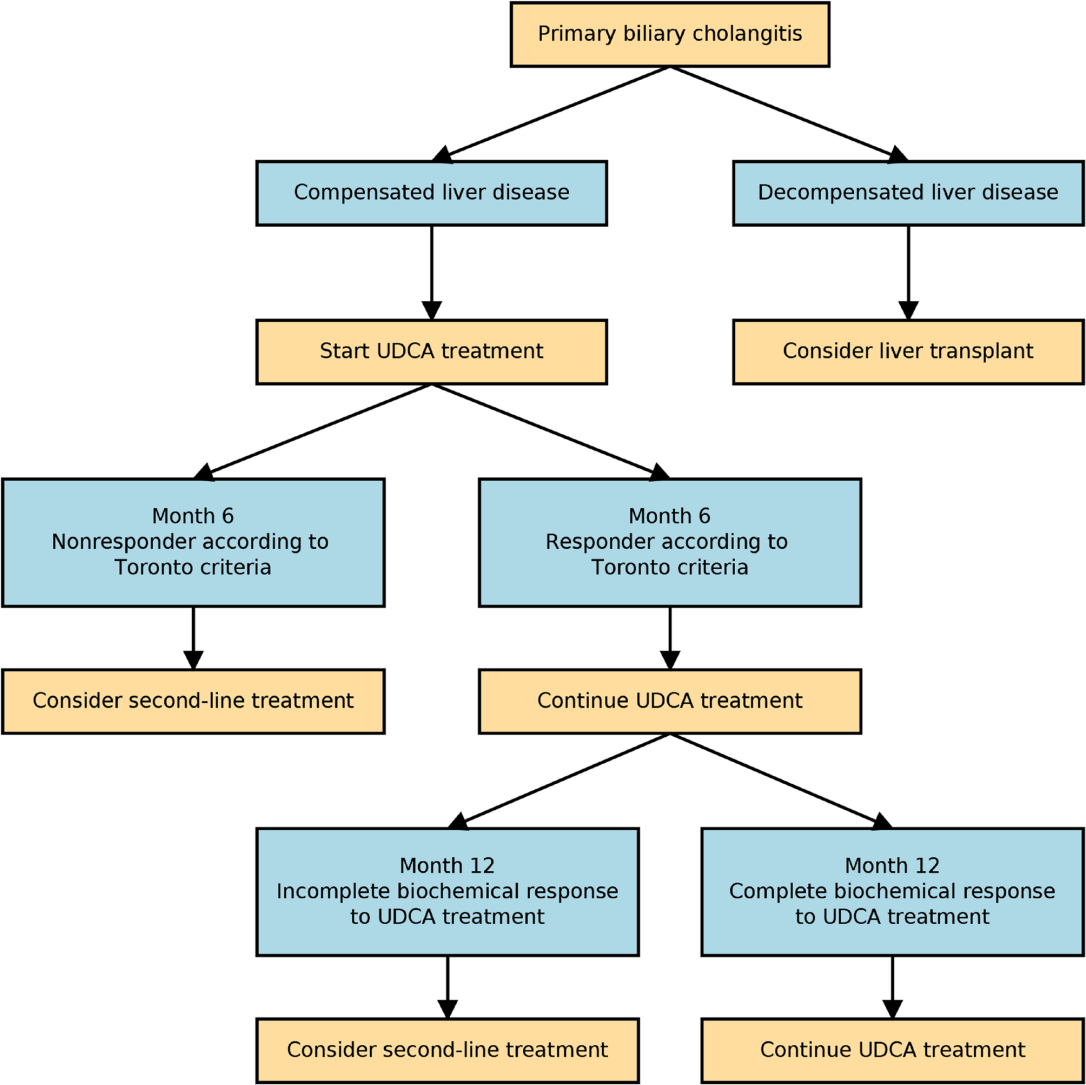
Recommendation of treatment algorithm for primary biliary cholangitis patients

Not only the drug selection, but also the timing of second-line treatment remains a crucial issue. Second line treatment is considered only after one year of treatment in most UDCA non-responders. However, delaying second line treatment may lead to worsening of the liver injury in some patients. Patients who did not achieve ALP ≤ 2.5×ULN, AST ≤ 2×ULN, and total bilirubin ≤ 1×ULN as soon as in one month of UDCA treatment had significantly higher 5-year mortality compared with responders according to the Chinese clinical trial [30]. This should lead to the consideration of second-line treatment in UDCA non-responders much earlier than after one year of treatment. No nonresponders according to Toronto criteria at month 6 achieved CBR at last check-up in our cohort. Nonresponders according to Toronto criteria at month 6 had a worse prognosis (higher number of hepatic decompensations, higher mortality) compared to responders. Therefore, for nonresponders according to Toronto criteria at month 6, second-line treatment should be considered after 6 months of UDCA treatment. We consider this result to be the most important finding of our study. Considering the findings of this study, we propose the modification of the indication of second-line treatment in PBC patients according to Fig. 2.

Although our study presented many very interesting findings, it has several limitations. The study design was retrospective. A small number of patients reached decompensation and mortality related outcomes which affected the statistical significance of the analysis of these outcomes (particularly all-cause and liver-specific mortality). The cohort of the patients was geographically restricted to a small region of Eastern Slovakia. The results of our observation will need to be validated in a larger multicenter study, preferably with a prospective design.

## Conclusion

Nonresponders to UDCA treatment have a worse prognosis compared to responders. Patients with complete biochemical response last check-up had very low chance of hepatic decompensation or liver-related death during follow-up. None of the patients who did not achieve biochemical response at month 6 by Toronto criteria, had CBR at last check-up. For non-responders by Toronto criteria at month 6, initiation of second line treatment should be considered after half a year of treatment.

## Data Availability

Availability of data and materials - we provided a table with our own research results in the section “Related files”.

## Abbreviations

AIH: Autoimmune hepatitis
ALP: Alkaline phosphatase
ALT: Alanine aminotransferase
AMA: Antimitochondrial antibodies
ANA: Antinuclear antibodies
anti-gp210: anti-glycoprotein-210 antibodies
anti-sp100: anti-sp100 nuclear antigen antibodies
anti-sp140: anti-sp140 nuclear antigen antibodies
AUROC: the total area under the receiver operating characteristic curve
AST: aspartate aminotransferase
bw: body weight
CBR: complete biochemical response
CRP: C-reactive protein
Dx: Diagnosis
EASL: European Association for the Study of the Liver
FGF-19: fibroblast growth factor 19
FXR: farnesoid X receptor
g: gram
GGT: gamma-glutamyltransferase
INR: international ratio
L: liter
M2: branched-chain alpha-keto acid dehydrogenase complex
mg: miligram
NOX 1: nicotinamide adenine dinucleotide phosphate oxidase 1
NOX 4: nicotinamide adenine dinucleotide phosphate oxidase 4
OCA: obeticholic acid
OR: odds ratio
PBC: primary biliary cholangitis
PSC: primary sclerosing cholangitis
POISE: phase III study of obeticholic acid in patients with primary biliary cirrhosis
PPAR: peroxisome proliferator-activated receptor
UDCA: ursodeoxycholic acid
ULN: upper limit of the normal
